# Phylogenetic identification of bacterial MazF toxin protein motifs among probiotic strains and foodborne pathogens and potential implications of engineered probiotic intervention in food

**DOI:** 10.1186/2045-3701-2-39

**Published:** 2012-11-27

**Authors:** Xianghe Yan, Joshua B Gurtler, Pina M Fratamico, Jing Hu, Vijay K Juneja

**Affiliations:** 1 Eastern Regional Research Center, Agricultural Research Service, U.S. Department of Agriculture, 600 E. Mermaid Lane, Wyndmoor, PA, 19038, USA; 2 Department of Mathematics and Computer Science, Franklin & Marshall College, P.O. Box 3003, Lancaster, PA, 17604, USA

**Keywords:** toxin-antitoxin module, probiotic cocktail, engineered probiotics, foodborne pathogens

## Abstract

**Background:**

Toxin-antitoxin (TA) systems are commonly found in bacteria and Archaea, and it is the most common mechanism involved in bacterial programmed cell death or apoptosis. Recently, MazF, the toxin component of the toxin-antitoxin module, has been categorized as an endoribonuclease, or it may have a function similar to that of a RNA interference enzyme.

**Results:**

In this paper, with comparative data and phylogenetic analyses, we are able to identify several potential MazF-conserved motifs in limited subsets of foodborne pathogens and probiotic strains and further provide a molecular basis for the development of engineered/synthetic probiotic strains for the mitigation of foodborne illnesses. Our findings also show that some probiotic strains, as fit as many bacterial foodborne pathogens, can be genetically categorized into three major groups based on phylogenetic analysis of MazF. In each group, potential functional motifs are conserved in phylogenetically distant species, including foodborne pathogens and probiotic strains.

**Conclusion:**

These data provide important knowledge for the identification and computational prediction of functional motifs related to programmed cell death. Potential implications of these findings include the use of engineered probiotic interventions in food or use of a natural probiotic cocktail with specificity for controlling targeted foodborne pathogens.

## Background

Foodborne illnesses continue to be an important public health concern in developing, as well as in developed countries, thus prevention of foodborne illness outbreaks through effective and novel interventions should be given priority. The U.S. Public Health Service has identified ten important foodborne pathogens causing human illnesses, including pathogenic strains of *Escherichia coli*, *Salmonella*, *Listeria, Clostridium botulinum*, *Shigella*, and *Campylobacter*, which are associated with more than 250 known foodborne diseases (http://www3.niaid.nih.gov/topics/foodborne/default.htm).

In addition, according to the World Health Organization (WHO), antibiotic overuse in food animal production is a major contributor to the emergence of antibiotic resistant foodborne pathogens [1]. The use of antibiotics in food animals for growth promotion and treatment disrupts the normal beneficial commensal bacterial microflora in the animal intestinal tract [2-6]. Recently, the human and chicken gut microbiome projects [7-12] have shed new light on the existence of a bacterial ‘phylogenetic core’ [13] consisting of a wide diversity of gastrointestinal bacteria by using new technologies such as next generation sequencing, 16S rRNA screens, metagenomics, and metaproteomics. Healthful, commensal bacteria found in the GI tract might be key members of known or potential probiotic strains revealed in this ‘phylogenetic core’, which may include *Bacillus clausii, Bacillus pumilus*, *Lactobacillus acidophilus, Lactobacillus reuteri, Lactobacillus rhamnosus GG* (ATCC 53103), *Bifidobacterium infantis, Saccharomyces boulardii*, *Lactobacillus ruminis*, *Lactobacillus johnsonii* str. NCC 533, and many others. These known probiotic strains have been used as dietary supplements, as treatments for illnesses caused by foodborne pathogens, and for disease prevention. Use of probiotic strains not only reduce invasion by bacterial pathogens, but also restore and maintain an optimal balance of healthy commensal bacteria in the human gut via production of antimicrobials [14-23].

One of the major mechanisms recognized as being responsible for apoptosis, or programmed cell death, and production of toxic metabolites in bacteria is through the regulation of a wide variety of bacterial toxin-antitoxin modules [24-26], such as MazE/MazF, a chromosomal toxin-antitoxin module [27-30], plasmid-encoded *par*D [31-33], *chpBIK*[26,34], *relBE*[35,36], and the PhoQ-PhoP system [37-40]. MazEF is one of the most well-studied toxin–antitoxin (TA) systems and has been found on the chromosomes of many bacteria. The MazF protein has been recently categorized as an endoribonuclease [41,42] or as a type of RNA interference enzyme [43]. The link between this TA system and quorum sensing has also been explored recently through a small pentapeptide (NNWNN) called the Extracellular Death Factor (EDF) [44]. This small peptide motif (such as NNWNN) is known to be an extra-cellular death factor in *E. coli* and other bacterial species. The necessity of an “extracellular death factor” (EDF) or “cell death factor” (CDF) via MazEF-mediated cell death is a population phenomenon requiring the activation of quorum-sensing (cell-to-cell signaling) peptides in bacteria. High cell density was found to be associated with elevated concentrations of EDF, and the presence of EDF resulted in MazF-induced cell death [44]. From a food safety and public health perspective, use of EDF or a similar strategy may be used in place of antibiotics, resulting in less usage of antibiotics. We also noticed in one very interesting study that the induction of toxin MazF and the use of antibiotic share a similar mechanism by inhibiting the transcription and/or translation of the MazE antitoxin [45].

It has been theorized that there is “one toxin for one antitoxin” and interestingly MazF, in some bacteria, exhibits a selective inhibition of ribosomes and mRNAs [43,46]. Numerous strains of probiotic bacteria, such as *Lactobacillus* spp., have been reported to produce antimicrobial agents [47], such as bacteriocins, that inhibit or kill closely-related species, or even different strains of the same species through the inhibition of transcription and translation by receptor binding. The antimicrobial activities of bacteriocins are due to a heterologous subgroup of ribosomally synthesized cationic peptides [48]. Nisin, a polycyclic antibacterial peptide 34 amino acid residues long, is one of the most studied bacteriocins and is produced by many strains of *Lactococcus lactis*. It has been approved by the FDA for use as a food preservative, and certain probiotic *Lactobacillus* strains have been thoroughly studied and evaluated *in vitro* and *in vivo*. For example*, L. reuteri* controls diarrhea in children and suppresses the growth and pathogenicity of harmful foodborne pathogens such as *Salmonella, E. coli*, *Staphylococcus*, and *Listeria*[49,50]. *L. casei* GG has been used to treat *Clostridium difficile* infections and to reduce intestinal permeability [51-54]. *L. reuteri* is known to produce a broad-spectrum antimicrobial agent, reuterin, composed of the natural metabolic compound 3-hydroxypropionaldehyde, which has been used on the surface of sausages to inhibit growth of harmful bacteria and fungi [15,16]. However, the molecular mechanisms underlying the effectiveness of individual probiotic strains have not been systematically studied and characterized. Several potential mechanisms of action, including their ability to generate diverse natural toxic metabolites, lactic acid, and other organic acids, enzymes, vitamins, and hydrogen peroxide, as well as antimicrobial peptides such as nisin, have been well-described [20].

The work reported herein explores the experimental antimicrobial possibilities and/or procedures for (1) expression of an engineered, stress-induced recombinant secreted fusion gene encoded by MazF and a small extracellular cell death factor (CDF) on the surface or extracellular space of recombinant probiotic bacteria or (2) for potential application of a cocktail of natural probiotic strains via experimental *in vitro* selection to control foodborne pathogens for improving the safety and quality of foods, as well as improving human health. The use of engineered probiotic strains or the natural probiotic cocktail consisting of mixed probiotic strain populations for targeting foodborne pathogens will potentially be able to selectively inactivate these pathogens, even in complex food matrices. Through gene/motif reshuffling [55,56] and computational molecular modeling, this engineered and secreted bacterial fusion protein, MazF-CDF, which contains an enterokinase (EK) cleavage site [57-60] for releasing active CDF and MazF after protein secretion, should have a narrow range of applicability, limited to inactivating only specific foodborne pathogens such as *E. coli* O157, *Salmonella, Listeria*, *Campylobacter,* and potentially other human pathogens*.* This pathogen specificity is due to the fact that the genetically-engineered MazF-CDF fusion protein could be modified by the inclusion of specific genomic DNA sequences from various commensal, as well as pathogenic bacterial strains, identified through DNA/protein structure and functional comparisons. Additionally, this engineered antimicrobial polypeptide and MazF will be non-toxic to beneficial (healthful/probiotic) bacteria, as well as to its host that expresses the protein/peptide. Moreover, these engineered and secreted CDFs and MazF proteins/peptides can easily pass through infectious foodborne pathogen cell barriers mediating cell death, and thus could potentially reduce the use of deleterious compounds such as antibiotics or other harmful chemicals in the feed and food industry.

## Results and discussion

### General genetic analysis of probiotics and foodborne pathogen genomes

The genomic information from selected probiotic strains and foodborne pathogens is shown in Table 1. It revealed little diversity in genomic GC-content in the *Bifidobacterium* genus, while showing an astonishing diversity in the GC-content among *Lactobacillus* species, ranging from 33 to 51.5%. It has been demonstrated that genomic GC-content is correlated with a number of factors [61], including genome size [62] from species such as *Lactobacillus*, which ranges from 1.8 to 3.4 Mb in length. This demonstrates that the GC-content and genome size of *Lactobacillus* genomes may have implications related to the biological complexity and adaptation of this genus, and could be due to the rate of recombination that has been extensively studied in the *E. coli* genome [63]. Knowledge of genetic diversity is fundamental to development of synthetic probiotic genomes and/or nucleic acid sequence reshuffling strategies. In this paper, we demonstrate that the MazF protein is a suitable candidate for the determination of genetic relationships within sets of MazF proteins in combination with with functional motif analysis.

**Table 1:** Genomic information of selected microorganisms considered as potential probiotic strains and some major food-borne pathogens used in this study

**Organism**	**Strain**	**Pathotype /or others**	**GenBank Accession /Assembly**	**GC (%)**	**Genome Size (~ Mb)**
*Campylobacter upsaliensis*	JV21	pathogenic	ASM18534v1	NA	1.6
*Lactobacillus coleohominis*	101-4-CHN	probiotic	ASM16193v1	41.3	1.7
*Campylobacter jejuni*	RM1221	pathogenic	NC_003912.7	30.3	1.8
*Lactobacillus johnsonii*	FI9785	probiotic	NC_013504.1	34.4	1.8
*Pediococcus pentosaceus*	ATCC 25745	probiotic	CP000422.1	37.4	1.8
*Streptococcus thermophilus* LMG	LMG 18311	probiotic	NC_006448.1	39.1	1.8
*Lactobacillus gasseri*	ATCC 33323	probiotic	NC_008530.1	35.3	1.9
*Lactobacillus sakei subsp. sakei*	23K	probiotic	NC_007576.1	41.3	1.9
*Lactobacillus delbrueckii subsp. bulgaricus*	ATCC 11842	probiotic	NC_008054.1	49.7	1.9
*Lactobacillus delbrueckii subsp. bulgaricus*	ATCC BAA-365	probiotic	NC_008529.1	49.7	1.9
*Leuconostoc citreum*	KM20	probiotic	ASM2640v1	38.9	1.9
*Bifidobacterium animalis subsp. Lactis*	Bl-04	probiotic	NC_012814.1	60.5	1.9
*Bifidobacterium animalis subsp. Lactis*	DSM 10140	probiotic	NC_012815.1	60.5	1.9
*Bifidobacterium animalis subsp. Lactis*	AD011	probiotic	NC_011835.1	60.5	1.9
*Lactobacillus amylovorus*	GRL1118	probiotics	ASM19411v1	38.0	2.0
*Lactobacillus johnsonii*	NCC 533	probiotic	NC_005362.1	34.6	2.0
*Lactobacillus acidophilus*	NCFM	probiotic	NC_006814.3	34.7	2.0
*Lactobacillus reuteri*	DSM 20016	probiotic	NC_009513.1	38.9	2.0
*Lactobacillus reuteri*	JCM 1112	probiotic	NC_010609.1	38.9	2.0
*Listeria monocytogenes*	FSL J1-208	pathogenic	ASM16843v1	37.7	2.0
*Lactobacillus salivarius*	UCC118	probiotic	NC_007929.1	33	2.1
*Lactobacillus helveticus*	DPC 4571	probiotic	NC_010080.1	37.1	2.1
*Leuconostoc mesenteroides subsp. mesenteroides*	ATCC 8293	probiotic	NC_008531.1	37.7	2.1
*Campylobacter concisus*	13826	pathogenic	NC_009802.1	39.3	2.1
*Lactobacillus fermentum*	IFO 3956	probiotic	NC_010610.1	51.5	2.1
*Bifidobacterium adolescentis*	ATCC 15703	probiotic	NC_008618.1	59.2	2.1
*Bifidobacterium bifidum*	PRL2010	probiotic	NC_014638.1	Na	2.2
*Bifidobacterium bifidum*	S17	probiotic	NC_014616.1	Na	2.2
*Lactobacillus amylovorus*	GRL 1112	probiotic	NC_014724.1	Na	2.2
*Lactobacillus brevis*	ATCC 367	probiotic	NC_008497.1	46.1	2.3
*Lactobacillus crispatus*	CTV-05	probiotic	ASM16588v1	37.1	2.3
*Bifidobacterium longum*	NCC2705	probiotic	NC_004307.2	60.1	2.3
*Bifidobacterium longum subsp. longum*	BBMN68	probiotic	NC_014656.1	Na	2.3
*Bifidobacterium longum subsp. infantis*	157F	probiotic	NC_015052.1	Na	2.4
*Bifidobacterium longum subsp. longum*	JCM 1217	probiotic	NC_015067.1	Na	2.4
*Lactococcus lactis subsp. lactis*	Il1403	probiotic	NC_002662.1	35.3	2.4
*Bifidobacterium longum*	DJO10A	probiotic	NC_010816.1	60.2	2.4
*Bifidobacterium longum subsp. longum*	JDM301	probiotic	NC_014169.1	Na	2.5
*Lactococcus lactis subsp. cremoris*	MG1363	probiotic	NC_009004.1	35.7	2.5
*Listeria grayi*	DSM 20601	pathogenic	ASM14899v1*	41.6	2.6
*Propionibacterium freudenreichii subsp. shermanii*	CIRM-BIA1	probiotic	NC_014215.1	Na	2.6
*Lactococcus lactis subsp. lactis*	KF147	probiotic	NC_013656.1	34.9	2.6
*Lactococcus lactis subsp. cremoris*	SK11	probiotic	NC_008527.1	35.8	2.6
*Bifidobacterium dentium*	Bd1	probiotic	NC_013714.1	58.5	2.6
*Bifidobacterium longum subsp. infantis*	ATCC 15697	probiotic	NC_011593.1	59.9	2.8
*Listeria monocytogenes*	EGD-e	pathogenic	NC_003210.1	38	2.9
*Lactobacillus casei str.*	Zhang	probiotic	NC_014334.1	40.1	2.9
*Lactobacillus casei*	ATCC 334	probiotic	NC_008526.1	46.6	2.9
*Lactobacillus paracasei subsp. paracasei*	ATCC 25302	probiotic	ASM15949v1	46.5	2.9
*Lactobacillus rhamnosus*	GG	probiotic	NC_013198.1	46.7	3.0
*Lactobacillus rhamnosus*	Lc 705	probiotic	NC_013199.1	46.7	3.0
*Lactobacillus casei*	BL23	probiotic	NC_010999.1	46.3	3.1
*Lactobacillus plantarum*	JDM1	probiotic	NC_012984.1	44.7	3.2
*Enterococcus faecalis*	V583	probiotic	NC_004668.1	37.4	3.3
*Lactobacillus plantarum*	WCFS1	probiotic	NC_004567.1	44.4	3.3
*Lactobacillus plantarum subsp. plantarum*	ST-III	probiotic	NC_014554.1	Na	3.4
*Bacillus selenitireducens*	MLS10	Bio agent	ASM9308v1	48.7	3.6
*Bacillus pumilus*	SAFR-032	probiotic	NC_009848.1	41.3	3.7
*Coprobacillus sp.*	29_1	Non-pathogenic	Coprobacillus_sp_29_1_V1	NA	3.8
*Vibrio cholerae*	M66-2	pathogenic	NC_012578.1	47.6	3.9
*Bacillus halodurans*	C-125	Non-pathogenic	ASM1114v1	43.7	4.2
*Bacillus amyloliquefaciens*	Y2	probiotic	ASM28439v1	45.9	4.2
*Clostridium difficile*	630	pathogenic	NC_009089.1	29.1	4.3
*Bacillus clausii*	KSM-K16	probiotic	NC_006582.1	44.8	4.3
*Shigella dysenteriae*	Sd197	pathogenic	ASM1200v1]	50.9	4.6
*Shigella flexneri*	8401	pathogenic	NC_008258.1	50.9	4.6
*Salmonella enterica subsp. arizonae serovar*	RSK2980	pathogenic	NC_010067.1	51.4	4.6
*Enterobacter sp.*	638	pathogenic	NC_009436.1	52.9	4.7
*Vibrio vulnificus*	CMCP6	pathogenic	NC_004459.2	46.7	5.1
*Escherichia coli*	E24377A	pathogenic	NC_009801.1	50.6	5.2
*Citrobacter rodentium*	ICC168	pathogenic	NC_013716.1	54.6	5.4
*Klebsiella variicola*	At-22	pathogenic	NC_013850.1	57.6	5.5
*Bacillus megaterium*	QM B1551	probiotic	ASM2582v1	38.0	5.5
*Escherichia coli* O157:H7	Sakai	pathogenic	NC_002695.1	50.5	5.6
*Bacillus thuringiensis serovar israelensis*	ATCC 35646	Bio agents	ASM16769v1	35.0	5.9
*Lachnospiraceae bacterium*	3_1_57FAA_CT1	Non-pathogenic	Lach_bact_3_1_57FAA_CT1_V1	NA	7.7

*: assembly accession.

### Ubiquitous existence of MazE/toxin, MazF

MazEF is a toxin-antitoxin (TA) module widely distributed among many bacterial species, including both foodborne pathogens and probiotic strains (Table 1, 2), such as *Lactobacillus acidophilus, Lactobacillus reuteri, Lactobacillus rhamnosus GG* (ATCC 53103), *Escherichia coli, Selenomonas sputigena, Enterobacter* spp.*, Campylobacter* spp.*, Citrobacter* spp.*, Thermoanaerobacter tengcongensis, Pelotomaculum thermopropionicum, Lactobacillus casei, Lactobacillus crispatus, Lactobacillus buchneri, Bifidobacterium longum subsp. infantis, Clostridium botulinum, Clostridium difficile, Vibrio* spp.*, Listeria* spp.*, Bacillus* spp.*, Klebsiella variicola, and Salmonella enterica.* Recent literature and computational analyses have shown the presence of many different types of TA modules in various localizations, e.g. some TA modules have been found within prophages, prophage-like elements, and other mobile genetic elements, such as plasmids [31-33]. Due to the existence of varying types of toxin-antitoxin modules and possible gene duplication events, in this paper we present the above one-to-one best matches of chromosomal-encoded *mazF* orthologs and homologs among 75 publically available genome sequences of foodborne pathogens and probiotic strains (Table 1), and the publically-available databases such as NCBI and the Uniprot database (http://www.uniprot.org/) in Table 2.

**Table 2:** **The genetic characterization of the transcriptional modulator *MazF*, a chromosomal cell death factor from potential probiotic strains and some major food-borne pathogens used in this study**

**Organism**	**Type of organisms**	**Strains**	**Protein accession (GenBank)**	**Gene annotation**
*E. coli*	Pathogen	EDL933	NP_289336.1	toxin ChpA
	Pathogen	Sakai	NP_311669.1	
	Antibiotic resistance	B185	ZP_06660634.1*	
*Enterobacter* sp.	pathogen	638	ABP60743.1*	transcriptional modulator of MazE/toxin, MazF
*Shigella dysenteriae*	pathogen	Sd197	YP_405833.1*	toxin ChpB
*Citrobacter rodentium*	pathogen	ICC168	YP_003366767.1*	Toxin component of the ChpB-ChpS toxin-antitoxin system
*Vibrio vulnificus*	pathogen	CECT4999=R99	YP_001393091.1*	growth inhibitor
*Listeria welshimeri* serovar 6b str.	pathogen	SLCC5334	YP_849071.1	PemK family transcriptional regulator
		FSL J1-208	ZP_05296226.1*	PemK family transcriptional regulator
*Listeria monocytogenes*		SLCC3954	YP_003464028.1	transcriptional regulator, PemK family
		FSL S4-120	ZP_07870171.1	toxin-antitoxin system, antitoxin component, MazF family
*Listeria seeligeri* serovar 1/2b str.				
*Listeria marthii*				
*Listeria grayi*	pathogen	DSM 20601	ZP_07054243.1*	MazF family toxin-antitoxin system protein
*Clostridium difficile*	pathogen	630	YP_001089981.1*	putative regulator of cell growth
		QCD-66c26	ZP_05273557.1	putative regulator of cell growth
		CIP 107932	ZP_05323891.1	putative regulator of cell growth
*Klebsiella variicola*	pathogen	At-22	YP_003438577.1*	transcriptional modulator of MazE/toxin, MazF pemK; protein PemK
		1_1_55	ZP_06548086.1	
*Salmonella enterica* subsp. arizonae serovar	pathogen	RSK2980	YP_001573441.1*	hypothetical protein SARI_04525
*Campylobacter showae*	pathogen	RM3277	ZP_05364729.1	addiction module antitoxin, RelB/DinJ family
*Campylobacter concisus*	pathogen	13826	YP_001466677.1	addiction module antitoxin
*Campylobacter jejuni* subsp.	pathogen	IA3902	ADC28395.1	prevent-host-death family protein
*Campylobacter upsaliensis*	pathogen	JV21	ZP_07894578.1*	ChpA/MazF transcriptional modulator
*Shigella flexneri* 2a str.	pathogen	301	NP_709188.1*	PemK protein
*Shigella flexneri* 5 str.		8401	YP_690766.1	growth inhibitor, PemK-like, autoregulated
*Cronobacter sakazakii*	pathogen	ATCC BAA-894	YP_001436394.1	bifunctional antitoxin/transcriptional repressor RelB
*Lactobacillus coleohominis*	probiotic	101-4-CHN	ZP_05553821.1*	regulatory protein
*Bacillus megaterium*	probiotic	QM B1551	YP_003560763.1*	endoribonuclease EndoA
		DSM 319	YP_003595503.1	
*Bacillus thuringiensis serovar israelensis*	probiotic	ATCC 35646	ZP_00740711.1*	MazF protein
		KBAB4	YP_001643128.1	MazF protein
*Bacillus weihenstephanensis*		AH1134	ZP_03233130.1	PemK family
*Bacillus cereus*				
*Coprobacillus sp.*	probiotic	29_1	ZP_08009764.1*	PemK family transcriptional regulator
*Enterococcus faecalis*	probiotic	V583	NP_814592.1	PemK family transcriptional regulator
		TX0102	EFQ11602.1*	
				Toxin-antitoxin system, toxin compoment, MazF
		TX2134	ZP_07557645.1*	
				Toxin-antitoxin system, toxin compoment, MazF
		HH22	ZP_03985009.1	
				PemK family transcriptional regulator
*Lactobacillus plantarum*	probiotic	WCFS1	NP_786238.1*	cell growth regulatory protein
*Lactobacillus rhamnosus*	probiotic	LMS2-1	ZP_04441477.1*	cell growth regulatory protein
		Lc 705	YP_003175134.1	transcriptional modulator of MazE/toxin, MazF
*Lactobacillus johnsonii*	probiotic	ATCC 33200	ZP_04007131.1*	PemK family growth inhibitor
*Lactobacillus crispatus*	probiotics	CTV-05	ZP_07789240.1*	ppGpp-regulated growth inhibitor
*Lactobacillus gasseri*	probiotic	ATCC 33323	YP_815460.1*	toxin-antitoxin system, toxin component, MazF family
		224-1	ZP_06262236.1	
		MV-22	ZP_07711551.1	
*Lactobacillus casei*	probiotic	BL23	YP_001986080.1	Cell growth regulatory protein
*Lactobacillus amylovorus*	probiotic	GRL1118	YP_005854914.1*	transcriptional modulator of MazE/toxin MazF
*Vibrio cholerae*	probiotic	1587	ZP_01950611.1*	transcriptional modulator of MazE/toxin, MazF
		NCTC 8457	ZP_01969676.1	
*Pediococcus pentosaceus*	probiotic	ATCC 25745	YP_805020.1*	toxin-antitoxin addiction module toxin component MazF (an endoRNAse)
*Bacillus amyloliquefaciens*	probiotic	Y2	YP_006327269.1*	Endoribonuclease
*Bacillus pumilus*	probiotic	SAFR-032	YP_001485694.1*	PemK family growth inhibitor endoribonuclease EndoA
		ATCC 7061	ZP_03054587.1	
*Lactobacillus sakei* subsp. sakai	probiotic	Sakei 23K	YP_396224.1	DNA-binding protein PemK family
*Leuconostoc mesenteroides* subsp. mesenteroides	probiotic	ATCC 8293	YP_819271.1*	toxin-antitoxin addiction module toxin component MazF
		ATCC 19254	ZP_03913232.1	PemK family growth inhibitor
*Leuconostoc mesenteroides* subsp. cremoris				
*Enterococcus faecalis*	probiotic	TX0102	EFQ11602.1*	toxin-antitoxin system, toxin component, MazF family
		TX0031	EFT96737.1	
*Bifidobacterium animalis subsp. lactis*	probiotic	HN019	ZP_02964075.1*	transcriptional modulator of MazE/toxin, MazF
*Bifidobacterium bifidum*	probiotic	NCIMB 41171	ZP_03645723.1	RelB antitoxin
*Bifidobacterium longum* subsp. longum	probiotic	JDM301	YP_003660624.1*	RelB antitoxin
		DSM 20213	ZP_06596513.1	toxin-antitoxin system protein
*Bifidobacterium breve*				
*Bifidobacterium bifidum*	probiotic	S17	YP_003938101.1	hypothetical protein with RelB antitoxin domain
*Lactobacillus casei*	Probiotic	ATCC 334	YP_807723.1	toxin-antitoxin addiction module toxin component MazF (an endoRNAse)
*Lactobacillus casei*	Probiotic	BL23	YP_001988635.1	Growth inhibitor
*Lactobacillus paracasei* subsp. paracasei	Probiotic	ATCC 25302	ZP_03963082.1*	PemK family transcriptional regulator
*Lactobacillus casei* str.	probiotic	Zhang	YP_003789562.1	toxin-antitoxin addiction module toxin component MazF
*Lachnospiraceae bacterium*	probiotic	3_1_57FAA_CT1	ZP_08609193.1*	hypothetical protein HMPREF0994_05199
*Leuconostoc citreum*	Probiotic	KM20	YP_001727503.1*	growth inhibitor
*Lactobacillus reuteri*	probiotic	DSM 20016	YP_001270863.1*	transcriptional modulator of MazE/toxin, MazF
		JCM 1112	YP_001841242.1	hypothetical protein LAR_0246
		MM2-3	ZP_03847756.1	PemK family growth inhibitor
*Streptococcus* sp. oral taxon 071 str.	probiotic	73H25AP	ZP_07458552.1*	ChpA/MazF transcriptional modulator
*Bacillus selenitireducens*	probiotic	MLS10	YP_003700699.1*	transcriptional modulator of MazE/toxin, MazF
*Bacillus halodurans*	probiotic	C-125	NP_244588.1*	ppGpp-regulated growth inhibitor (ChpA/MazF)
*Enterococcus faecium*	probiotic	DO	ZP_05714797.1*	PemK family protein
		TX0133a04	ZP_07845579.1	toxin component, MazF family
		TX0133C	ZP_07850444.1	toxin component, MazF family

**Figure 1:**
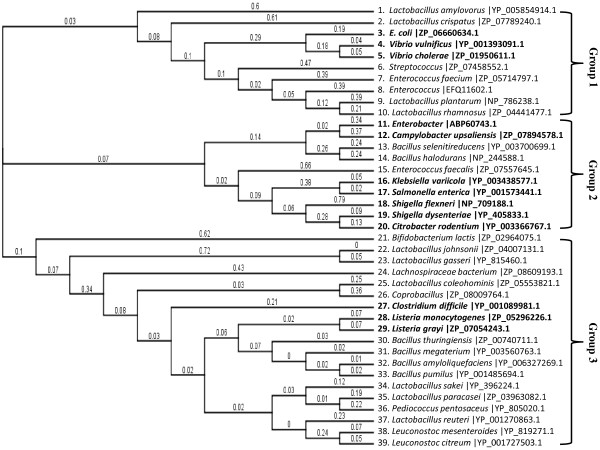
**Phylogenetic analyses of Enterobacteriaceae based on MazF (toxin), a growth inhibitor.** The NCBI accession numbers are shown after each taxon name.

*: data are presented in Figure 1.

### Phylogenetic analyses and cluster analysis of MazF/antitoxin protein, a growth inhibitor

The phylogenetic tree in Figure 1 displays the phylogentic relationships of many well-recognized genera within *Enterobacteriaceae*, including several important foodborne pathogens such as pathogenic *E. coli*, *Salmonella*, *Listeria*, and *Campylobacter,* as well as some major probiotic strains. To build a phylogenetic tree from the data in Table 2, the amino acid sequences of all the MazF or growth inhibitor proteins were analyzed using the Geneious software package v5.5.7 with Neighbor-joining (NJ) method by applying ClustalW for sequence alignment (Figure 1). Three main clusters (viz., groups 1, 2, and 3) are given in Figure 1. At least two representatives of potential probiotic strains are listed for each group, depending on the complexity of groups. In group 1, the potential probiotic, non-pathogenic strains, such as *Lactobacillus amylovorus, Lactobacillus crispatus, Streptococcus, Enterococcus faecium, Enterococcus, Lactobacillus plantarum,* and *Lactobacillus rhamnosus,* are grouped with the foodborne pathogens *E. coli, Vibrio vulnificus, and Vibrio cholerae.* In group 2, the foodborne pathogens *Enterobacter*, *Campylobacter upsaliensis*, *Klebsiella variicola*, *Salmonella enterica*, *Shigella flexneri*, *Shigella dysenteriae*, and *Citrobacter rodentium* were shown to be genetically closer to *Bacillus selenitireducens, Bacillus halodurans, and Enterococcus faecalis* In group 3, some probiotic strains, such as the *Bacillus, Lactobacillus, Leuconostoc,* and the *Bifidobacterium* genera are categorized along with other major foodborne pathogens, such as *Clostridium difficile, Listeria monocytogenes, Listeria grayi,* and an emerging foodborne pathogen *Pediococcus pentosaceus*.

### Phylogenetic identification of bacterial toxin MazF protein motifs and the relationship between gene structure and phylogenetic classification

**Figure 2:**
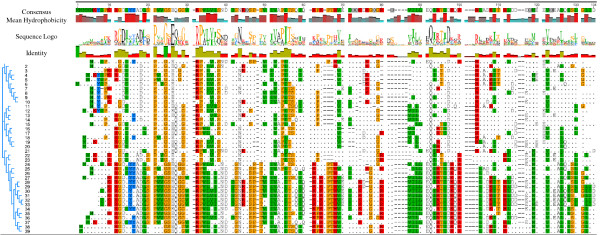
**ClustalW output (default settings) for the aligned amino acid sequences of MazF.** The pdf images of the alignments were generated using Geneious v5.5.7. Conserved amino acids are highlighted in colors; the numbers (1 to 39) in this Figure correspond to the taxon names in Figure 1 .

In Figure 2, it is shown that the number of candidate motifs are slightly different in each group, but with a high degree of amino acid sequence variability within conserved “hot spots” between groups 1, 2, and 3. To determine which motifs are the best candidates for the engineered MazF construction (discussed in next section) in each individual group, recent studies [43] suggest that the N-terminal (the first 80 amino acids in Figure 2) of the MazF protein are the most suitable motifs. The other motifs in the C-terminal might be referred to as ‘incorrect’ motifs. There are three criteria for evaluating the remaining motifs as “incorrect and without biological significance”, and this is related to: the mean hydrophobicity, identity, and the gene structure. In Figure 2, in the analysis for the phylogenetic identification of bacterial Toxin MazF protein motifs, the mean hydrophobicity sequence and identity were computed and compared for each sequence; it is interesting to find that all the compared MazF proteins have a higher degree of mean hydrophobicity, identity, and more conserved gene structure among several conserved amino acid regions, particular in the N-terminal. The selection of potential functional MazF motifs is discussed in the next section. The functional importance of the mean hydrophobicity has also been discussed to involve mRNA and protein degradation and slower translation of mRNA for disordered proteins (http://employees.csbsju.edu/hjakubowski/classes/ch331/protstructure/olprotfold.html).

### Bacterial probiotic cocktail strains

It is important to note that the benefits of probiotics are strain-specific [64,65]. A commercial product, VSL#3, developed by Sigma-Tau Pharmaceuticals, Inc., provides a mixture of probiotic bacteria (*B. breve, B. infantis, B. longum, L. acidophilus, L. bulgaricus, L. casei, L. plantarum,* and *Streptococcus thermophilus*) to help protect GI tract integrity [66]. In this study, the bacterial probiotic cocktail strains we propose would be comprised of all representatives of groups 1, 2, and 3 shown in Figure 1*.* The principal basis behind the composition of these probiotic cocktail strains is the assumption that a combination of organisms might be more effective than the application of a single strain, which potentially could suppress many foodborne pathogens, such as the *E. coli* and vibros in Group 1, several foodborne pathogens, such as *Enterobacter, Klebsiella variicola, Salmonella enterica, Shigella flexneri, Shigella dysenteriae, Citrobacter rodentium*, and *Campylobacter upsaliensis,* (one of the most common *Campylobacter* strain found in people with diarrhea in Group 2), *Clostridium difficile, Listeria monocytogenes,* and *Listeria grayi,* in Group 3 of Figure 1. Table 1 shows an astonishing diversity in the genomic GC-content and genome size among *Lactobacillus* species and their diverse distribution in all groups within Figure 1, which indicates a potential to further identify other closely-related *Lactobacillus* species (not listed in Table 1) into the three previously-described groups*.* The hypothesis underlying our approach is that probiotic strains found within the same group with foodborne pathogens will have a reasonable degree of genetic and molecular phylogenetic compatibility and could bridge a relationship similar to a “symbiosis” of entities, including exchanging toxin/antitoxin molecules among the probiotic and pathogenic strains. *Lactobacillus* species are known to produce antimicrobial substances, including bacteriocins, lactic acid, and hydrogen peroxide. The MazEF toxin component may provide a basis for bacterial growth inhibition within the same group (Figure 1 and 2). Therefore, this toxin-antitoxin module may have great potential to inhibit the growth of potentially-pathogenic bacteria through a possible competitive exclusion due to selective inhibition [46]. Figures 1 and 2 list all possible cocktails of these probiotic strains. In reality, a foodborne outbreak is more likely to be associated with one particular foodborne pathogen in particular foods. For example, there is a low incidence of *Campylobacter* in ground beef and pork, while *Campylobacter* is the major foodborne pathogens associated with poultry, therefore, a single food-borne pathogen with the application of mixed probiotics will be considered initially.

### Molecular recombination techniques: construction of genetically engineered synthetic probiotic strains

**Figure 3:**
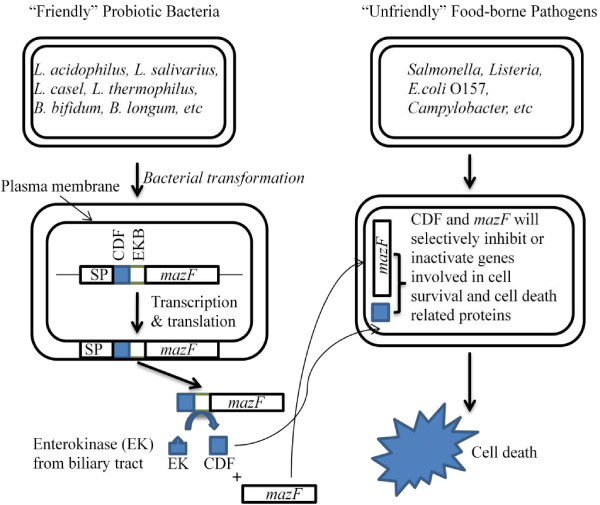
**A Schematic representation of the engineered, secreted CDF and *MazF*-mediated cell death during food processing and storage.** SP: promoter, CDF: cell death factor/peptide, EKB: enterokinase binding site.

Figure 3 details an engineered probiotic strain bearing a recombinant plasmid containing a stress-induced promoter, followed by an in-frame gene fusion accomplished by fusing an appropriate signal peptide, a functional cell death peptide/factor (CDF), an enterokinase (EK) binding site, and a genetically-modified engineered MazF gene*,* which will be constructed and transformed into the probiotic bacteria. In this recombinant probiotic strain, environmental stress, relevant to food environments will trigger gene expression of this fusion protein under an environmental stress-inducible promoter. The signal peptide directs the encoded fusion protein to the extracellular space of the engineered probiotic strains. The signal peptide depleted fusion proteins will be cleaved by a biliary tract enterokinase directly into the digestive system to release the functionally-active CDF and MazF. This event will occur based on the ability of probiotic strains to form stable, non-infectious biofilm-type aggregates, which may attach with other bacteria, including foodborne pathogens, in the urogenital and intestinal tracts [22]. These active species-specific CDF and MazF proteins will bind and pass through “unfriendly” bacterial cell surface barriers into the cytoplasm. These processed CDFs and MazF proteins will selectively inactivate and/or inhibit proteins involved in cell survival and induce the synthesis of more cell death-related proteins with activity against foodborne pathogens, eventually controlling, inhibiting, or inactivating “unfriendly” cells. Although antitoxin MazE could reverse the bacteriocidal effect of the overexpressed MazF, MazE cannot impede the downstream cascade already initiated by MazF at early stages of the MazF-mediated cascade [42].

The overall hypothesis for this experiment is graphically presented in Figure 3. The engineered MazF gene sequence will be designed based on the genomic sequences of all publically-available foodborne pathogenic strains by using reasoned random gene biosynthesis and/or genuine gene reshuffling to rapidly combine functions and properties of parental genes for the development of improved gene specificity and generality, molecular modeling (transcription factor binding site identification, etc.), and systems biology technologies/tools.

## Conclusions

Survival of foodborne pathogens in cultures or in animal GI tracts may be very genus- or species-specific. Data presented in this paper can be explored to develop effective intervention strategies applied directly during food processing and preparation, as well as in the animal feed supply, which may lead to an overall reduction in use of antibiotic growth promoters (AGP) throughout the world. Recent research in molecular biology and genomics has provided potential applications of probiotic strains as dietary supplements, which could replace AGP in animal diets or as biotherapeutic agents in cases of antibiotic-associated diarrhea in travelers and in childhood diarrhea and other bacterial gastrointestinal illnesses. Experiments relating to this potential probiotic application may reveal a further greater range of potential benefits. For many of these potential benefits, current research is limited, and only preliminary results are available. All effects can only be attributed to the individual strain(s) tested. Testing of a specific supplement may not be extrapolated to benefits from any other strain of the same species, and testing results do not imply that comparable benefits will be imparted from other LAB (or other probiotics). In this study, we have computationally explored several potential intervention strategies to control foodborne pathogens, either by using a cocktail of probiotic strains or an engineered probiotic strain.

The inhibition of pathogens by probiotic strains is mainly due to the production of antibacterial peptides [67], the release of short-chain fatty acids, or reduction of the pH within the lumen [68,69] by the production of organic acids or by decreasing pathogen adherence to intestinal epithelial cells [70]. Therefore, the benefits of probiotics could be very strain- or species-specific, and probiotic strains may rely on different mechanisms to suppress growth, attachment, or other metabolic processes, inherent to pathogenic bacteria. Moreover, the optimal effects of probiotic strains may involve the simultaneous use of more than one strain. Our contention in this paper is not experimental proof but, rather, a clear scientifically-backed hypothesis in the form of a detailed accompanying method that multi-probiotic strain composites with diverse genetic backgrounds may complement [71] one another as vectors of competitive exclusion and, therefore, could maximize the potential to inhibit an array of common foodborne pathogens [72] in the gastrointestinal tract of humans or livestock, as well as in foods and animal feed.

The use of probiotic bacteria with the ability to produce CDFs and engineered MazF to selectively inactivate pathogens is a novel approach to controlling pathogens in foods, and possibly treating human infections. A number of studies suggest that this project could have practical significance and be a potentially new approach for the development of novel and cost-effective food safety intervention technologies for the control of foodborne pathogens and improving public health [73-75].

The approaches described above represent a first attempt to describe a systematic approach or method to test the hypothesis that “friendly” bacteria can be used to inactivate or inhibit pathogens in food based on expression of MazF. There are many specific cell death factors that may be associated with bacterial programmed cell death and multi-cellular behavior mechanisms in foodborne pathogens. Through computational modeling, remodeling, genetic recombination, or further gene reshuffling, and exploring experimental approaches, it may be possible to evaluate and elucidate more effective CDFs and MazF to be used for controlling foodborne pathogens, which will ultimately result in a reduction in the use of antimicrobial compounds in humans and animals, as well as during food processing and storage.

## Materials and methods

### Genomic sequences

All of the genome and gene sequences examined in this study (Table 1 &2) are available in GenBank (http://www.ncbi.nlm.nih.gov/genbank/GenbankOverview.html).

### Identification and analysis of bacterial toxin-antitoxin modules, MazE/MazF

The prediction accuracy of the best chromosomal-encoded MazF orthologs among relatively distinct genome strains is critical for the performance of molecular phylogenetic analysis. We used a sequential BLAST workflow based on pairwise comparison by applying either an *E. coli* programmed cell death toxin MazF (Genbank accession: ZP_06660634.1), an endoribonuclease MazF, a MazF protein from *Vibrio cholerae* (ZP_01950611.1), or a PemK family transcriptional regulator protein (ZP_05296226.1) from *Listeria monocytogenes* to perform a BLASTP homology search of these combined protein sequences with all 75 publically available foodborne pathogen and probiotic strain genomic sequences (presented in Table 1) publically available databases such as NCBI and Uniprot database (http://www.uniprot.org/). BLAST search results were used to list MazF or MazF-like proteins from 75 different strains being represented as 39 different species (Table 2).

### Phylogenetic analysis and computational identification of phylogenetic motifs of the MazF protein

In order to identify potential functional motifs of MazF proteins, phylogentic analyses of MazF proteins were conducted by applying the embedded multiple sequence alignment ClustalW program in the Geneious software package v 5.5.7 [76,77] with the Neighbor Joining method. ClustalW output for the aligned amino acid sequences and the pdf images of the alignments were generated using the Geneious software package v 5.5.7.

## Competing interests

Mention of trade names or commercial products in this publication is solely for the purpose of providing specific information and does not imply recommendation or endorsement by the U.S. Department of Agriculture. USDA is an equal opportunity provider and employer.

## Authors’ contributions

XY conceived the study. XY and JH performed the bioinformatics study and XY wrote the paper. All authors read and approved the manuscript.
